# From genes to organs: physiological responses of European chub (*Squalius cephalus*) to chronic PFAS pollution

**DOI:** 10.3389/ftox.2025.1654272

**Published:** 2025-09-26

**Authors:** Sara Pacchini, Laura Drago, Martina Cortese, Giacomo Vanzan, Elisabetta Piva, Shaghayegh Kholdihaghighi, Andrea Barbarossa, Anisa Bardhi, Sophia Schumann, Chiara Fogliano, Andrea Bottacin-Busolin, Paola Irato, Andrea Marion, Gianfranco Santovito

**Affiliations:** ^1^ Department of Biology, University of Padova, Padova, Italy; ^2^ Department of Environmental Sciences, Informatics and Statistics, Ca’ Foscari University of Venice, Mestre, Italy; ^3^ Department of Veterinary Medical Sciences, University of Bologna, Bologna, Italy; ^4^ Department of Biology, University of Naples Federico II, Naples, Italy; ^5^ Department of Industrial Engineering, University of Padova, Padova, Italy

**Keywords:** PFAS, chronic stress, freshwater fish, catalase, glutathione peroxidases

## Abstract

Per- and polyfluoroalkyl substances (PFAS) are widespread anthropogenic contaminants known to the scientific community for their persistence and toxicity. Our research aims to evaluate the effect of chronic environmental exposure to PFAS on the antioxidant system of *Squalius cephalus*. In particular, to better understand how various components of the antioxidant system act together to counteract the adverse effects of PFAS, in the present study we evaluate the gene expression and enzymatic activity of two selenium-dependent glutathione peroxidases (namely GPx-1 and GPx-4) and the catalase, in the two major organs involved in the accumulation and detoxification from pollutants, including PFAS, i.e. liver and caudal kidney. Fish were sampled from four sites in the Veneto region with different concentrations of total dissolved PFAS. To better assess the acclimatisation to the environment, the morphological characteristics of the fish were also examined, as well as the development of organs, through the calculation of some somatic indices. Lipid accumulation was demonstrated histologically in both the liver and caudal kidney, which likely occurs to attenuate the high reactivity of PFAS toward protein content in these organs. The results demonstrate how *Squalius cephalus* can survive chronic PFAS exposure through cellular and systemic physiological responses.

## Highlights

• PFAS exposure activates the H_2_O_2_ screening system, mainly in the caudal kidney.
• Se-GPx and CAT act complementarily in response to PFAS in the caudal kidney.
• Lipids accumulate in the liver and caudal kidney of *Squalius cephalus* exposed to PFAS.
• Immune response is probably induced in the spleen at low PFAS concentrations.

## 1 Introduction

Among the anthropogenic contaminants, per- and polyfluoroalkyl substances (PFAS) have garnered the attention of the scientific community and public opinion in the last decade due to their widespread use and high persistence and toxicity in the environment (Cousins et al., 2020), which can also increase in relation to Climate change (Bortot et al., 2025; Cunha et al., 2025). As they are used in a wide range of fields, from carpeting to dental floss (Glüge et al., 2020), PFAS have a global distribution and are even present in Antarctica (Xie et al., 2022). The amphiphilic structure of PFAS, consisting of a polar functional group and a hydrophobic fluorinated tail, endows these substances with a high affinity for proteins, such as liver fatty acid-binding proteins and serum albumin. As a result, PFAS can bioaccumulate within organisms and be transferred along the trophic chain (Ferrario et al., 2022; Lewis et al., 2022; Pietropoli et al., 2025). Regarding human health, some of the main studied effects of exposure to PFAS include alteration of immune function and interference with the efficacy of vaccines, liver and kidney diseases, including cancer, diabetes due to alteration of lipids and insulin concentration, infertility and growth failure (Bonato et al., 2020; Fenton et al., 2021). The manufacture and use of the most well-known PFAS, such as perfluorooctanoic acid (PFOA) and perfluorooctane sulfonate (PFOS), have been restricted in many countries precisely because of their significant potential risks to human health and the environment (Louisse et al., 2020).

In 2013, a significant industrial PFAS contamination of surface water and groundwater was discovered in the Veneto region, Italy (Giglioli et al., 2023). According to annual monitoring data from the Regional Agency for Environmental Prevention and Protection of Veneto (ARPAV) (ARPA Veneto, 2025), the presence of PFAS in freshwater streams continues to be assiduous, making it necessary to investigate the ability of organisms to cope with the stress induced by prolonged exposure to PFAS (Vaccari et al., 2024).

Among the species currently subjected to this anthropogenic impact is *Squalius cephalus*, also known as European chub, a pelagic freshwater fish abundant throughout Europe, commonly used as a target species in ecotoxicological studies (Molbert et al., 2019; Sunjog et al., 2019; Staszny et al., 2021). *S. cephalus* specimens typically live 15 years, with males becoming fertile at 2–4 years and females at 4–6 years (Nyeste et al., 2024).

Our previous study showed that PFAS contamination in rivers of the Veneto region led to a significant increase in transcript levels of *gpx-4* and *sod-2*, two genes encoding mitochondrial antioxidant enzymes, in the liver of *S. cephalus* and another species, *Padogobius bonelli*. The results suggested that oxidative stress, which could lead to lipid peroxidation due to chronic PFAS exposure, was controlled in this organ by the antioxidant system at the mitochondrial level (Piva et al., 2022). The adequate protection of lipids from PFAS was also confirmed by analyses performed on the kidney of *S. cephalus*, in which, however, protein oxidation was detected also at low PFAS concentrations (Pacchini et al., 2025b). In this case, the gene encoding the isoform 4 of peroxiredoxins (*prdx-4*) appeared not to be involved in the antioxidant defences, probably carried out by other components of the antioxidant system. Further analyses also revealed high stress levels in the muscle and blood of *S. cephalus* and *P. bonelli* (Schumann et al., 2024), underscoring the need to extend the investigation of responses against PFAS to other organs and genes.

Glutathione peroxidases (GPxs) and catalase (CAT) act mainly in H_2_O_2_ scavenging. GPxs reduce organic and inorganic peroxides to hydroxyl compounds, using glutathione (GSH) or other equivalents as reducing factors. Among the known eight GPx isoforms in humans, three are monomeric, i.e. GPx-4, -7 and -8, and the remaining five are homotetrameric. GPx-3, -6 and -7 are the only extracellular isoforms; the others are intracellular. Among the latter, isoform 4 is the only one present in mitochondria. The first four isoforms and GPx-6 are selenium (Se)-dependent, as they present a selenocysteine residue encoded by the stop codon TGA (Brigelius-Flohé and Maiorino, 2013; Ferro et al., 2020; Pacchini et al., 2025a). While GPx-4 can reduce complex fatty acid, phospholipid, and cholesterol hydroperoxides, including those inside membranes, GPx-1, -2, and -6 are supposed to react with less complex, soluble, but also small fatty acid hydroperoxides (Trenz et al., 2021). Unlike GPxs, CAT exists as a single isoform in animals that can be distinguished into three classes based on its sequence and structural differences. The monofunctional heme-containing enzyme is the most widespread CAT in all aerobic organisms. The bifunctional CAT-peroxidase belongs to the second class, which also contains the heme group but is relatively less abundant in nature and closely related to plant peroxidases. The third class belongs to the Mn-containing CAT group that lacks the heme group (López et al., 2024). CAT is a tetrameric protein that can cleave two H_2_O_2_ molecules into two water molecules and one oxygen molecule, acting mainly within peroxisomes. CAT has been reported to be implicated in mutagenesis, inflammatory conditions, and the suppression of apoptosis, all of which are known to be associated with oxidative stress conditions (Nandi et al., 2019).

The present work aims to gain a deeper understanding of how the components of the antioxidant system interact to counteract the adverse effects of PFAS in *S. cephalus* on the liver and caudal kidney. *S. cephalus* specimens were collected from four rivers characterised by different concentrations of total dissolved PFAS, from <10 ng/L (control site) to over 1,000 ng/L (highly-polluted site). The antioxidant molecular markers considered in this study were GPx isoforms 1 and 4 and CAT. Both gene expression and enzyme activity were assessed in the liver and caudal kidney, organs known for their roles in the bioaccumulation of pollutants in fish and detoxification processes, as they filter contaminants from the bloodstream and metabolise them for elimination, primarily *via* urine (Collard et al., 2018; Macorps et al., 2022).

Additionally, to better study the acclimatisation to the environment, the morphological characteristics of the fish were also examined, as well as the development of their organs, calculating the hepatosomatic index (HSI), Fulton’s condition factor (FCF) and the spleen somatic index (SSI). Finally, liver and caudal kidney tissues were chemically analysed to verify if PFAS accumulation increased proportionally to the increase in PFAS concentration in the environment, and were examined by hematoxylin and eosin staining to highlight cellular damage and lipid content.

## 2 Materials and methods

### 2.1 Sampling activity

Fifteen specimens of *S. cephalus*, 25 ± 6 cm in length, were sampled by electrofishing (authorised by decree of the director of the Agri-environment, Planning, and Management of Fish and Wildlife Hunting of the Veneto Region, n. 384 of 17 May 2024) in April 2024, from four selected freshwater streams. The sampling sites are drainage basins in agricultural areas of the Vicenza province (Veneto region, northeastern Italy) with different concentrations of total dissolved PFAS (i.e. PFBA, PFPeA, PFHxA, PFHpA, PFOA, PFNA, PFDA, PFUnDA, PFDoDA, PFBS, PFHxS, PFOS), and with other dissolved pollutants, such as metals and pesticides volatile organic compounds, at negligible concentration (<10 ng/L), according to ARPAV periodic monitoring data measured over 2022–2023 (ARPA Veneto, 2025). Long-term industrial emissions of PFAS in the province of Vicenza were first identified in 2013 and attributed to the Rimar-Miteni plant, located in Trissino. Wastewater from the facility was discharged into the Agno-Fratta-Gorzone river system, leading to widespread downstream dispersion of PFAS through a complex hydrological network (Castiglioni et al., 2015). The Rimar-Miteni plant ceased operations and was declared bankrupt in November 2018. Following this, the Veneto Region launched extensive environmental monitoring programs to assess PFAS levels in surface and groundwater (World Health Organization Regional Office for Europe, 2017).

Considering the limit of quantification (LOQ) of 500 ng/L for total PFAS established by the Directive (EU) 2020/2184 of the European Parliament and of the Council of 16 December 2020, on the quality of water intended for human consumption, we named the four sampling sites as follows: control site (total PFAS <10 ng/L), low-polluted site (total PFAS at 10–500 ng/L), medium-polluted site (total PFAS at 500–1,000 ng/L), and highly-polluted site (total PFAS >1,000 ng/L) (Supplementary Table S1). The study area, represented in Supplementary Figure S1, was georeferenced in QGIS (v. 3.22) using the Gauss–Boaga coordinate system (EPSG: 3003/3004) (Supplementary Table S1), in accordance with ARPAV data standards: the control site, located in Grumolo delle Abbadesse (VI), corresponds to the Roggia Moneghina (Bacchiglione river basin); the low-polluted site, located in Sovizzo (VI), corresponds to the Fosso Brenta (Bacchiglione river basin); the medium-polluted site, located in Lonigo (VI), corresponds to the Scolo Togna (Fratta-Gorzone river basin); the high-polluted site, located in Trissino (VI), corresponds to the Torrente Poscola (Fratta-Gorzone river basin). The medium-polluted site is historically known to receive industrial discharges from the former Rimar–Miteni plant, the highly-polluted one is where the Rimar–Miteni plant was located.

The sampled fish, after being euthanised with an overdose of essential clove oil prepared in water-soluble form by dilution with ethyl alcohol (concentration 7 μL/L), were stored on ice and immediately transferred to the Department of Biology of the University of Padua to proceed with the dissection and the removal of organs.

### 2.2 Tissue preparation

Before dissection, all specimens were weighed and measured for total body length. The liver, caudal kidney, intestine, gills, gonads, spleen, heart, brain, white muscle, and tail were removed from the fish. The sex of the individuals, all of fertile age, was assigned through macroscopic observation of the gonads. The liver and spleen were weighed to calculate the HSI and SSI indices, respectively. All the dissected organs were frozen in liquid nitrogen and stored at −80 °C for future molecular, biochemical and chemical analyses. In addition, for histological analyses, part of the dissected organs was fixed in Karnovsky’s solution (4% paraformaldehyde, 0.1% glutaraldehyde in 0.2 M cacodylate buffer containing 1.7% NaCl, pH 7.4), dehydrated in 80% ethanol and stored at −20 °C. In the present study, the molecular and fat accumulation responses to PFAS-induced stress were analysed in the liver and caudal kidney of *S. cephalus*.

### 2.3 Primer design

Primers for selection (100–200 nt) and quantification of *cat* and *gpx-1* expressions by quantitative real-time PCR (qRT-PCR) (Supplementary Table S2) were designed on the coding DNA sequences (CDSs) of the respective genes of *Pimephales promelas*, collected from NCBI database (accession number for *cat*: XM_039669564.1; accession number for *gpx-1*: XM_039688995). *Pimephales promelas* is a fish of the Cyprinidae family, phylogenetically close to *S. cephalus*. After checking primer parameters by IDT Oligo Analyser tool (https://www.idtdna.com/calc/analyzer), primers for *cat* and *gpx-1* were synthesised by Merck Life Science S.r.l. (Milan, Italy). Primers for *gpx-4* and *gapdh* (housekeeping gene), reported in Supplementary Table S2, were the same as those used in our previous study (Piva et al., 2022).

### 2.4 Total RNA extraction, cDNA synthesis and sequencing

Total RNA was extracted from the liver and caudal kidney tissues of eight specimens from each sampling site, using PRImeZOL (Canvax) as a lysis buffer according to the manufacturer’s protocol. Total RNA extracted from liver tissues was purified with 8 M lithium chloride to remove carbohydrate contaminants (Bakiu et al., 2022a); further purification of all RNA samples was performed with the RQ1 RNase-Free DNase (Promega) kit to remove any possible genomic contamination. Total RNA concentration and purity were assessed with the Nanodrop ND-1000 spectrophotometer (Thermo Fisher Scientific), and its integrity and the absence of genomic DNA contamination were verified through electrophoresis on a 1% agarose gel.

Reverse transcription from 1 μg of each extracted total RNA was performed with the BiotechRabbit cDNA Synthesis Kit, which includes Oligo (dT) primer for selecting poly(A) tailed mRNA.

The obtained cDNAs were checked by qualitative PCR performed with 2X YourTaq PCR Master Mix (BiotechRabbit) kit, and the primers reported in Supplementary Table S2, on a SimpliAmp Thermal Cycler (Applied Biosystems). Amplicons corresponding to *cat*, *gpx-1*, *gpx-4*, and *gadph* were visualised on a 1.5% agarose gel, and amplicons obtained for *cat* and *gpx-1* were gel-purified with Wizard SV Gel and PCR Clean-Up System (Promega) kit and validated by sequencing, performed by Eurofins genomics (Europe Shared Services GmbH, Ebersberg, Germany). The obtained *cat* and *gpx-1* sequences were checked with the BLAST tool (https://blast.ncbi.nlm.nih.gov/Blast.cgi).

### 2.5 qRT-PCR analysis

Eight cDNAs (biological samples) from the liver and caudal kidney of *S. cephalus*, for each sampling site, were amplified with primers reported in Supplementary Table S2, according to qPCRBIO SyGreen Mix Separate-ROX (PCR Biosystems) kit instructions. ROX concentration in the reaction mix, used as a quencher, was adjusted to 100 nM. qRT-PCR analysis of *cat*, *gpx-1*, *gpx-4*, and *gapdh* (housekeeping gene) was performed on a 7500 Real-Time PCR Systems (Applied Biosystems) thermal cycler according to the following amplification thermal profile: 95 °C for 2 min, 38 cycles at 95 °C for 20 s and 60 °C for 1 min (amplification plot), and, finally, 95 °C for 15 s, 60 °C for 1 min, 95 °C for 15 s, and 60 °C for 15 s (melting curve). First, primer amplification efficiency was verified by absolute quantification using scalar-diluted cDNAs. Each cDNA (<100 ng) was run three times (technical triplicate), and the melting profile was analysed to verify the absence of genomic contamination.

Relative values obtained by qRT-PCR were used in the 2^−ΔΔCT^ Pfaffl mathematical model (Pfaffl, 2001). Transcription levels were normalised to those of the housekeeping gene to compensate for variations in the amounts of cDNA.

### 2.6 Preparation for biochemical analysis

Liver and caudal kidney tissues were homogenised in a buffer containing 10 mM Tris-HCl (pH 7.6), 1 mM EDTA, 1 mM dithiothreitol, 0.5 M sucrose, and 0.15 M KCl, with the Kinematica Polytron PT-MR 3000 Homogeniser. After centrifugation at 13,000 × *g* for 1 h at 4 °C, cell-free extracts were analysed for total soluble proteins (mg/mL) by the Folin phenol reagent method (Lowry et al., 1951). To do this, a calibration curve was first built using scalar concentrations of bovine serum albumin as standards. Total protein concentrations were used to normalise the data of CAT and Se-GPx activities.

### 2.7 CAT activity assay

CAT activity was determined using Aebi’s method (Aebi, 1984). The decrease in absorbance in 1 mL reaction mix, containing 50 mM potassium phosphate buffer (pH 7.0), 10 mM H_2_O_2_, and 30 μL of the cell-free extract, was measured at 240 nm for 1 min and expressed as units of CAT/mg proteins. One unit of CAT was defined as the amount of enzyme catalysing the scavenging of 1 µmol of H_2_O_2_/min.

### 2.8 Se-GPx activity assay

Livingstone’s method was applied to measure the Se-GPx activity in cell-free extracts (Livingstone et al., 1992). 900 μL of the mixture were prepared, containing 100 µL of cell-free extract in 50 mM potassium phosphate buffer (pH 7.0), 1 mM EDTA, 1 mM sodium azide, 0.2 mM NADPH, 1 U/mL of glutathione reductase, and 1 mM GSH; 100 µL of 20 mM H_2_O_2_ were added to initiate the reaction. The decrease in NADPH concentration was recorded at 340 nm in 5 min. Data were expressed as units of Se-GPx/mg proteins, where one unit of Se-GPx was defined as the amount of enzyme catalysing the oxidation of 1 µmol GSH/min.

### 2.9 Hematoxylin and eosin staining

Dehydration of liver and caudal kidney of fish (biological duplicate) from control and highly-polluted sites was completed in ethanol 100% and, before proceeding with embedding in Paraplast X-TRA (Tyco Kendall), organs were treated with xylene for 1 h. 5 μm of histological sections were obtained with a Leica Reichert-Jung 2040 Autocut microtome and left to adhere to microscope glass slides (Menzel-Glaser, ThermoFisher Scientific). Tissue sections were deparaffinised in xylene and gradually rehydrated through a descending ethanol series. Sections were stained in hematoxylin solution (Sigma-Aldrich) for 1 min and subsequently rinsed under running tap water for 15 min. Staining was continued with 1% eosin Y (Sigma-Aldrich) in 80% ethanol for 30 s. Dehydration of the sections was rapidly completed in 100% ethanol, followed by a brief treatment with xylene. Finally, the sections were mounted with Eukitt (Electron Microscopy Sciences) and examined under the Olympus CX31 light microscope to assess morphological changes and lipid content.

### 2.10 Somatic indices

PFAS effects on the physiology of *S. cephalus* were also evaluated by calculation of the following somatic indices (Singh and Reddy, 1990; Nilsson et al., 1996; Martínez et al., 2004):

$$\text{HSI }\left(g/g\right)=\left(\text{liver weight}/\text{total body weight}\right)\times 100$$

$$\text{SSI }\left(g/g\right)=\left(\text{spleen weight}/\text{total body weight}\right)\times 100$$

$$\text{FCF }\left(g/\text{cm}\right)=\left[\text{total body weight}/{\left(\text{total}\;\text{length}\;\text{of}\;\text{the}\;\text{individual}\right)}^{3}\right]\times 100$$

### 2.11 UHPLC-MS/MS analysis

Pure PFAS analytical standards, including the ^13^C-labeled standards ^13^C_2_-PFOA and ^13^C_4_-PFOS, were obtained from Wellington Laboratories (Guelph, Ontario, Canada). Ultrapure water was freshly produced on each day of analysis using a Sartorius system (Milan, Italy). PFAS quantification was performed by ultra-high-performance (UHP) liquid chromatography (LC) coupled with tandem mass spectrometry (MS/MS).

Fish tissues were pre-treated following the approach described by Gasparini et al. (2024), with slight modifications. Briefly, 50 µL of an internal standard mixture, comprising 100 ng/mL of ^13^C_2_-PFOA and ^13^C_4_-PFOS in water, was added to 5 g of fish tissue (biological duplicate/triplicate when possible) to assess bioaccumulation. Then, 2.5 mL of 200 mM sodium hydroxide and 10 mL of methanol were added to each sample, which was then homogenised using an Ultraturrax (IKA, Staufen, Germany). After homogenisation, 150 µL of 4 M hydrochloric acid was added and samples were agitated on a vortex mixer, then centrifuged at 10,000 × *g* for 10 min. A solid-phase extraction using Oasis GCB/WAX cartridges (6 mL, 150 mg, 30 μm; Waters, Milford, MA, United States) was performed to extract target analytes from tissue cell-free extracts. Cartridges were conditioned with 4 mL of methanol and 4 mL of water. Then, 8 mL of tissue supernatant or 50 mL of water sample was loaded, and cartridges were washed with 4 mL of 2% formic acid in water, followed by 4 mL of methanol. Target analytes were eluted with 3 mL of 1% ammonium hydroxide in methanol. The eluates were evaporated under a gentle nitrogen stream at 45 °C, reconstituted in 200 µL of an 80:20 (v/v) water:methanol solution, and transferred into polypropylene vials for UHPLC-MS/MS analysis.

The system consisted of an Acquity UPLC binary pump (Waters, Milford, MA, United States), equipped with the perfluorinated compounds isolation kit to avoid background contamination. Chromatographic separation was performed using a Waters Acquity UPLC BEH C18 column (50 × 2.1 mm, 1.7 μm), maintained at 40 °C. The mobile phase consisted of 5 mM ammonium acetate in water and methanol at a flow rate of 0.3 mL/min under programmed conditions. The UPLC was interfaced to an XEVO TQ-S Micro triple quadrupole mass spectrometer (Waters, Milford, MA, United States), set in negative electrospray ionisation mode with a capillary voltage of −0.50 kV. The source and desolvation temperatures were 150 °C and 500 °C, respectively; the cone gas was set at 50 L/h, and the desolvation gas at 900 L/h, while argon was used as the collision gas. The specific transitions monitored for each analyte, along with the corresponding cone voltage (CV) and collision energy (CE) values, are reported in Supplementary Table S3. Data were acquired and processed using MassLynx 4.2 software (Waters, Milford, MA, United States).

During each day of analysis, method validation was carried out directly in the sample matrix (a pooled homogenate of various tissues) using the matrix standard addition approach. Calibrators (range: 0.005–5.0 μg/kg) and quality control (QC) samples at four concentration levels (0.005, 0.2, 1.0, and 5.0 μg/kg), containing all target analytes, were freshly prepared to assess the method’s performance in terms of specificity, linearity, precision, and accuracy. Peak area ratios between analytes and internal standards were plotted against the corresponding concentrations, and a linear least squares regression model was applied. All calibrators had to be within ±15% of their nominal values, and the resulting correlation coefficient (*r* ^2^) had to be ≥0.99. All QC samples had to confirm the good accuracy (within ±15%) and precision (coefficient of variation <15%) of the method for all analytes. The limit of quantification (LOQ) for all PFAS was set at 5 μg/kg, and the limit of detection (LOD) was defined as 2 μg/kg. The recovery was within 93%–107% for all compounds. The use of isotopically labelled internal standards minimised matrix effects and further improved the reliability of quantification, confirming the robustness and validity of the method.

Finally, specificity was proved by the absence of chromatographic signals at the retention times of the target analytes in blank samples, even after the injection of the highest-concentration calibrators.

### 2.12 Statistical analysis

For all analyses, data were expressed as means ± standard deviations for each sampling site, with eight biological samples (n = 8), and statistically compared using the JASP program (Version 0.19). Although the number of specimens analysed is limited, it remains consistent with sample sizes commonly employed in stress physiology research and is sufficient to generate meaningful results (Weber et al., 2020; Solé et al., 2021; Atli et al., 2024).

After checking the variance’s uniformity with Levene’s test (p > 0.05), a one-way variance analysis (ANOVA) was applied, followed by Tukey’s test to analyse statistically significant (p < 0.05) differences among the means.

## 3 Results

### 3.1 Qualitative PFAS bioaccumulation analysis

Due to the limited availability of tissue samples, PFAS concentrations could not be measured in the organs of the single specimens, but it was necessary to pool tissues from eight fish per site. This approach, although reducing replication and preventing formal statistical analysis, is a common and accepted practice in bioaccumulation studies when sample sizes or tissue amounts are limited (Smith et al., 2018; Jones and Lee, 2020). Therefore, the results presented here should be considered as indicative rather than statistically conclusive.

As shown in Supplementary Table S4, PFOS was the predominant PFAS accumulated in both liver and caudal kidney of *S. cephalus*, followed by perfluorodecanoic acid (PFDA). In the liver and caudal kidney, the accumulation of these two PFAS progressively increased from the control site to the highly polluted site. In particular, PFOS concentrations in the liver increased nearly tenfold, while PFDA levels rose more than fivefold.

In the caudal kidney, PFOS concentrations at the highly polluted site were approximately five times higher than those at the control site, whereas PFDA showed a sixfold increase.

### 3.2 Transcription levels of *cat, gpx-1*, and *gpx-4* in the liver and caudal kidney

**FIGURE 1:**
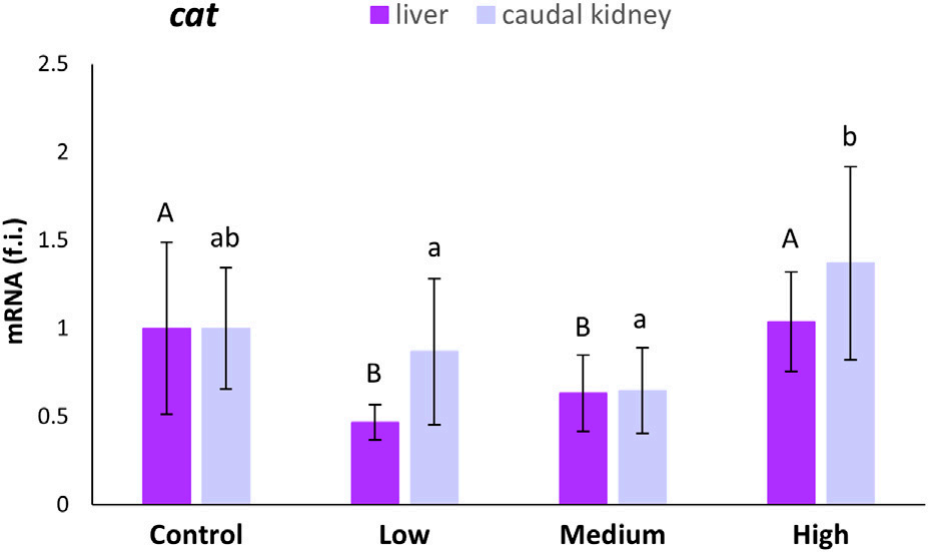
Relative expression levels (fold induction, f.i.) of the *cat* mRNA in the liver and caudal kidney of *S. cephalus* specimens from the four sampling sites (control, low-polluted, medium-polluted, highly-polluted). Transcription levels for the polluted sites were normalised with respect to those for the control site (mean = 1). Different letters refer to statistically significant differences among the means (p < 0.05).

Statistically significant decreases (53% and 37%, respectively) in *cat* transcription levels were observed in the liver of *S. cephalus* only for the low- and medium-polluted sites compared to levels measured for the control site (Figure 1). No statistically significant variation in *cat* mRNA expression levels was observed in the caudal kidney of fish from the polluted sites with respect to fish from the control site.

**FIGURE 2:**
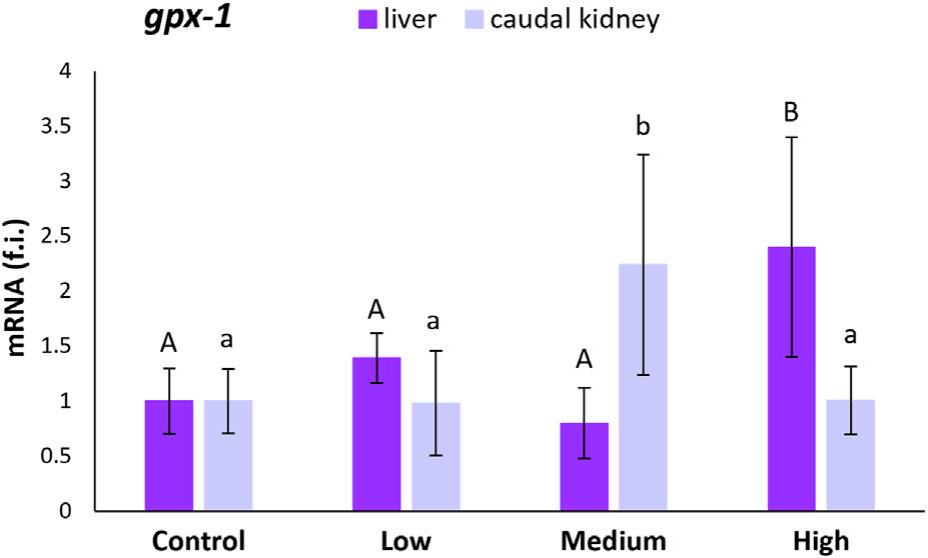
Relative expression levels (fold induction, f.i.) of *gpx-1* mRNA in the liver and caudal kidney of *S. cephalus* specimens from the four sampling sites (control, low-polluted, medium-polluted, highly-polluted). Transcription levels for the polluted sites were normalised with respect to those for the control site (mean = 1). Different letters refer to statistically significant differences among the means (p < 0.05).

The *gpx-1* transcription levels were statistically higher in the liver of specimens from the highly-polluted site than in the liver of fish from the control site (140%) and the other two polluted sites (Figure 2). The same was observed for the medium-polluted site in the caudal kidney. For this site, *gpx-1* transcription levels increased by 124% with respect to levels in the kidney of fish from the control site (Figure 2).

**FIGURE 3:**
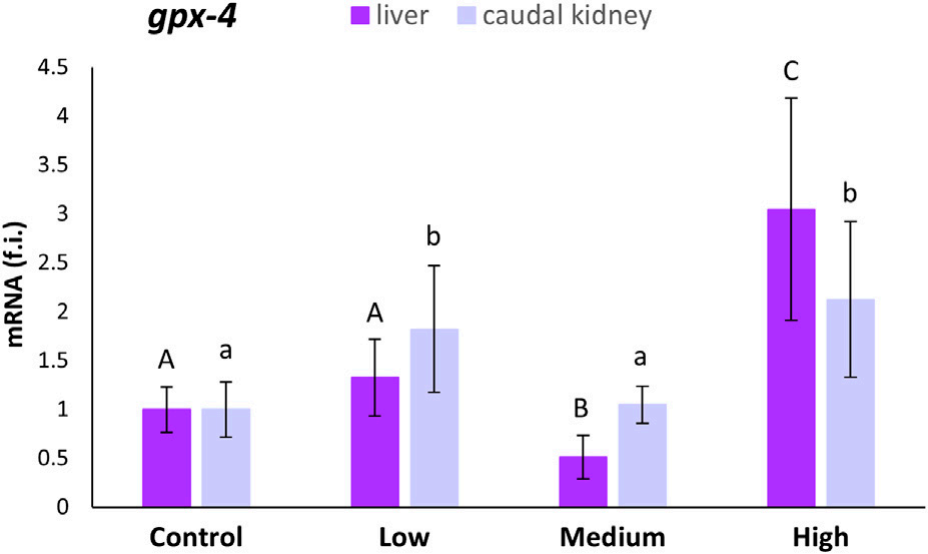
Relative expression levels (fold induction, f.i.) of *gpx-4* mRNA in the liver and caudal kidney of *S. cephalus* specimens from the four sampling sites (control, low-polluted, medium-polluted, highly-polluted). Transcription levels for the polluted sites were normalised with respect to those for the control site (mean = 1). Different letters refer to statistically significant differences among the means (p < 0.001).

The *gpx-4* mRNA expression levels in the liver statistically decreased (47%; p < 0.05) in specimens from medium-polluted ones with respect to fish from the control site. On the contrary, the liver samples from the highly-polluted site showed an increase of 204% (Figure 3). In the case of the caudal kidney, statistically significant increases (p < 0.05) in *gpx-4* expression levels, with respect to levels referred to the control site, were observed only for the low- (82%) and highly-polluted (112%) sites (Figure 3).

### 3.3 CAT and Se-GPx activities in the liver and caudal kidney

**FIGURE 4:**
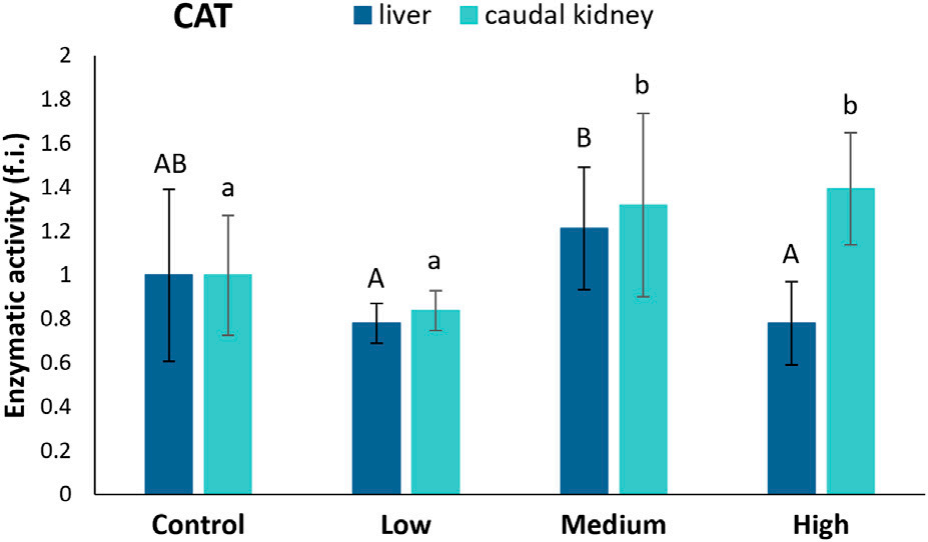
CAT activity (fold induction, f.i.) in the liver and caudal kidney of *S. cephalus* specimens from the four sampling sites (control, low-polluted, medium-polluted, highly-polluted). Active protein levels for the polluted sites were normalised with respect to those for the control site (mean = 1). Different letters refer to statistically significant differences among the means (p < 0.05).

CAT activity in the liver of *S. cephalus* remained relatively constant in the specimens from the control and polluted sites (Figure 4). Inside the caudal kidney, the CAT activity statistically increased in specimens from the medium- (32%) and highly-polluted (39%) sites compared to fish from the control site (Figure 4).

**FIGURE 5:**
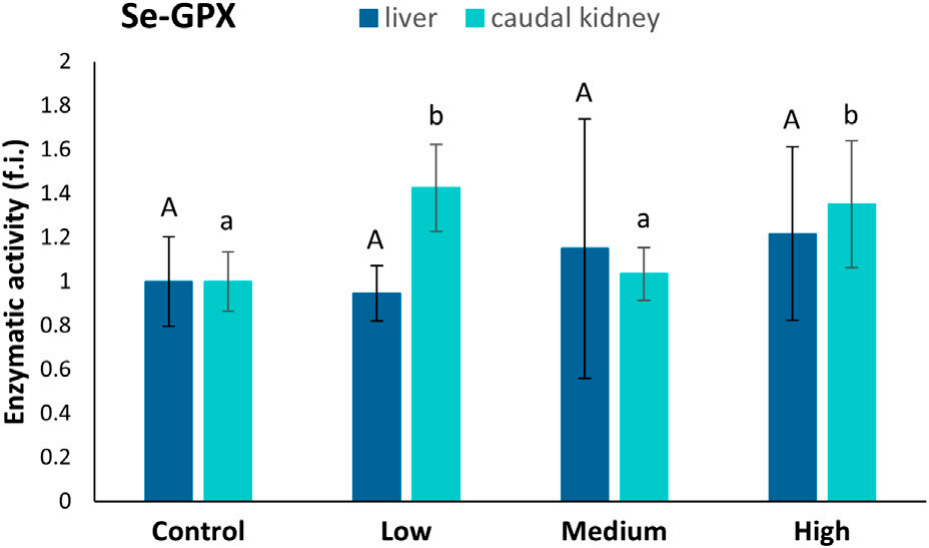
Se-GPx activity (fold induction, f.i.) in the liver and caudal kidney of specimens from the four sampling sites (control, low-polluted, medium-polluted, highly-polluted). Active protein levels for the polluted sites were normalised with respect to those for the control site (mean = 1). Different letters refer to statistically significant differences among the means (p < 0.05).

No statistically significant differences were found among the four sampling sites regarding the Se-GPx levels in the liver (Figure 5). In the caudal kidney, the activity of Se-GPx showed statistically significant increases in fish from the low- (42%) and highly-polluted (35%) sites compared to fish from the control one (Figure 5).

### 3.4 Morphological damages and fat content within the liver and caudal kidney

**FIGURE 6:**
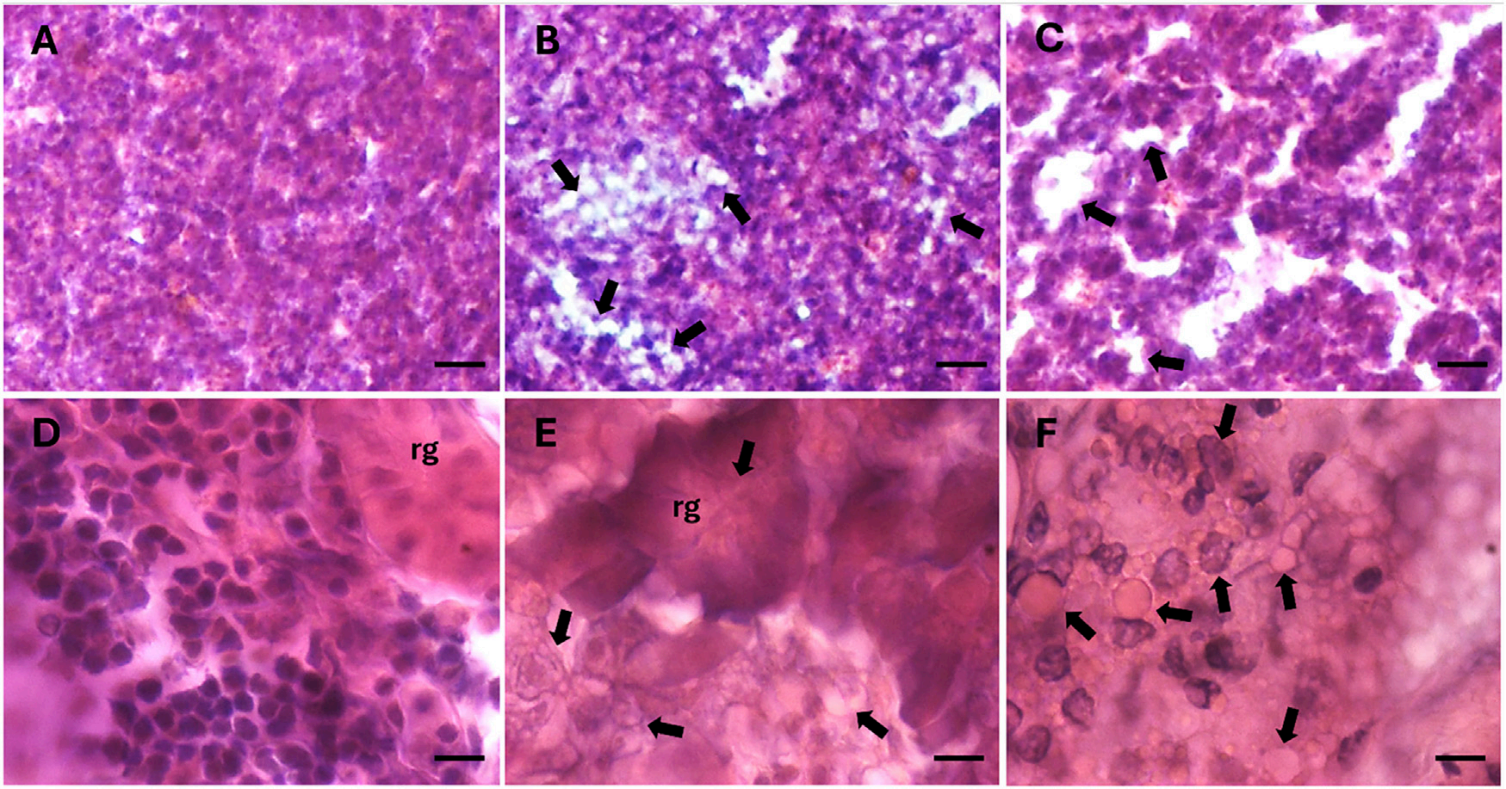
Histological sections of liver **(A–C)** and caudal kidney **(D–F)** of *S. cephalus* from the control site **(A and D)** and the highly PFAS-contaminated site **(B–C and E–F)**. Arrows indicate lipid accumulation within tissues. Nuclei are stained in dark purple, cytoplasm in fuchsia. rg: renal glomerulus. Scale bar: 100 µm **(A–C)**, 10 µm **(D–F)**.

Hematoxylin and eosin staining revealed structural alterations in the renal glomeruli of fish from the highly-polluted site, which appeared contracted compared to those from control fish (Figure 6E vs. Figure 6D). In specimens from the polluted site, both the glomeruli and infiltrating erythrocytes in the kidney exhibited lipid vacuolisation, not only in the cytoplasm (Figures 6E,F), but also within the nuclei (Figure 6F). Additionally, a marked release of lipid content outside the cells was observed in the liver tissue of fish from the highly polluted site (Figures 6B,C), in contrast to the liver of control fish (Figure 6A).

### 3.5 SSI, HSI, and FCF somatic indices

**FIGURE 7:**
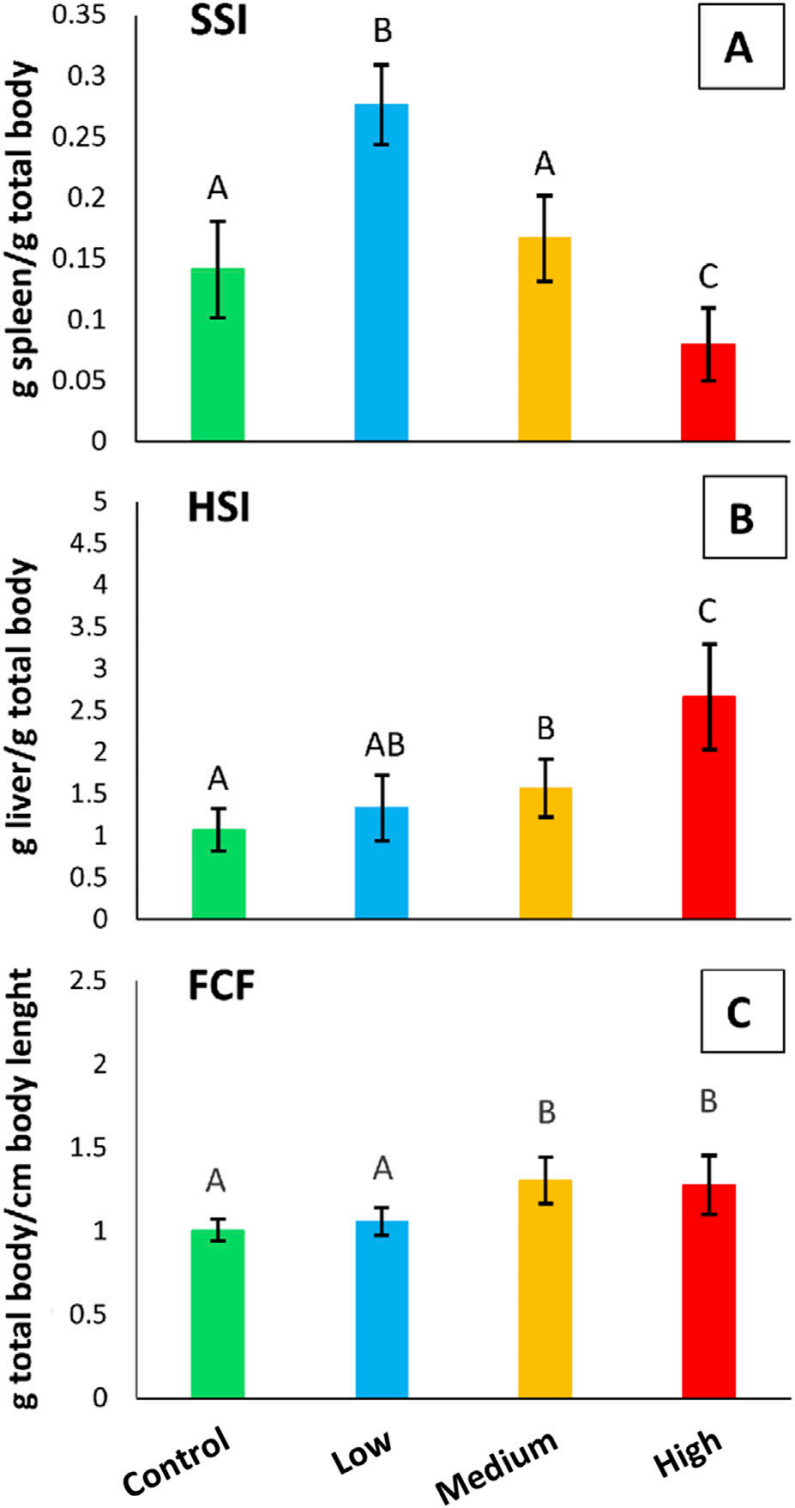
**(A)** SSI, **(B)** HSI, and **(C)** FCF somatic indices referred to *S. cephalus* specimens from the four sampling sites (control, low-polluted, medium-polluted, highly-polluted). Different letters refer to statistically significant differences among the means (p < 0.05).

Figure 7 shows the SSI, HSI, and FCF indices calculated for *S. cephalus* from the four sampling sites. The SSI index showed a statistically significant increase (95%; p < 0.05) in the specimens from the low-polluted site with respect to those from the control one. Conversely, fish from the highly-polluted site showed a statistically significant decrease (43%; p < 0.05) in the SSI index when compared to fish from the control site (Figure 7A). The HSI index showed statistically significant increases (p < 0.05) for fish from the medium- (47%) and highly-polluted (149%) sites, compared to fish from the control one (Figure 7B). Similar to HSI, the FCF index showed statistically significant increases (p < 0.05) for fish from the medium- (30%) and highly-polluted (27%) sites compared to fish from the control one (Figure 7C).

## 4 Discussion

Most research on PFAS focuses on human health (Biggeri et al., 2024), while little is known about their effects on freshwater fauna. Given that water is the primary polluted matrix in Veneto (Biggeri, 2024; Vaccari et al., 2024), investigating the role of chronic exposure to PFAS on the physiology of *S. cephalus*, an endemic and cosmopolitan fish species in the region, may be fundamental to better understanding their impact on river ecosystems.

Previous studies have shown that *S. cephalus* can activate a range of physiological responses at both cellular and systemic levels in response to PFAS exposure (Piva et al., 2022; Schumann et al., 2024; Pacchini et al., 2025b). In the present study, in addition to evaluating three somatic indices to obtain a general overview of the wellbeing of the sampled *S. cephalus* specimens, we investigated the antioxidant responses of this freshwater fish that counteract oxidative stress induced by H_2_O_2_ overproduction. To this aim, we examined the induction of CAT and Se-GPx, both at transcriptional and enzymatic activity levels, in the liver and caudal kidney, two organs involved in the accumulation and detoxification of xenobiotics.

Regarding the HSI, we observed a clear increase in liver size in fish from medium- and highly-polluted sites. This finding is consistent with previous evidence linking liver enlargement in *S. cephalus* to total PFAS concentrations (Piva et al., 2022) and, in our case, specifically to PFOS and PFDA, the predominant compounds detected in our analyses. Hepatic PFOS levels reached 55.40 μg/L and 72.78 μg/L at the medium- and highly-polluted sites, respectively, while PFDA concentrations were 3.31 μg/L and 21.67 μg/L. These concentrations are in line with the liver being a primary PFOS target organ (Wang et al., 2022) and fall within ranges associated with hepatic steatosis in fish and rodents chronically exposed to PFAS (Attema et al., 2022; Cheng et al., 2016). Such steatosis, driven by stress-induced lipid accumulation in hepatocytes, may reduce PFAS interactions with cellular proteins by modifying the hydrophilicity of the intracellular environment and promoting non-specific binding to lipids (Yoo et al., 2009; Qin et al., 2023). Moreover, PFAS can disrupt lipid metabolism through activation of PPARα and PPARγ, further enhancing lipid deposition (Li et al., 2024; Wang Q. et al., 2023; Johnson et al., 2024), while lipid droplets may confer a protective effect by sequestering reactive lipid peroxidation products and limiting oxidative damage (Martinez et al., 2023). The liver is not the only organ affected by weight increases following PFAS exposure, as indicated by the FCF index, which in our study also revealed higher total body weight in *S. cephalus* from medium- and highly-polluted sites.

Our histological analyses revealed lipid accumulation not only in the liver but also in the caudal kidney. While there is limited evidence in fish regarding renal lipid deposition linked to PFAS, human studies have associated chronic kidney disease with PFAS exposure (Conway et al., 2018), and this condition is known to cause lipid dysmetabolism and lipid droplet accumulation in the kidney (Mitrofanova et al., 2023). Supporting this hypothesis, we observed downregulation of *prdx-4* mRNA expression in the caudal kidney of *S. cephalus* chronically exposed to PFAS (Pacchini et al., 2025b). Since this antioxidant enzyme inhibits lipid accumulation in cells (Yamada and Guo, 2018), its reduced expression may reflect a cellular response facilitating lipid deposition in the kidney. Similarly, Kim et al. (2023) demonstrated that reduced antioxidant enzyme activity promotes lipid droplet formation under chemical stress.

The SSI index results showed the highest values in fish from the low-polluted site and the lowest in those from the highly-polluted site. The spleen is particularly interesting because it plays a crucial role in regulating the immune system. Previous studies on mice exposed to PFOS have evidenced a decrease in spleen weight, which was associated with an increase in liver weight (Wang et al., 2011), suggesting an adverse effect of this PFAS on the immune system. Spleen immunosuppression was also highlighted in zebrafish exposed to PFOA (Zhong et al., 2020). The spleen plays a crucial role in erythropoiesis, the process of producing red blood cells. However, in fish from medium- and highly-polluted sites, this function may be predominantly carried out by the head kidney (Chen et al., 2013; Witeska, 2013). The increase in spleen weight observed at the low-polluted site is likely due to enhanced erythrocyte production or activation of the immune response. This response may be necessary when evaluating prolonged exposure to PFAS, even at low concentrations. Based on these considerations, it is plausible that the spleen enlarges at the low-polluted site as a primary defence mechanism against the effects of PFAS. When these effects become too excessive for the organism (medium- and highly-polluted sites), the spleen goes under stress, decreasing its mass. Therefore, the spleen appears to play a secondary role in defending against PFAS when their concentrations are high, leaving the liver and the body’s general lipid accumulation to deal with the harmful effects of PFAS.

We observed a limited activation of the studied antioxidant enzymes in the liver. No change in CAT activity is consistent with previous findings in rainbow trout liver (Tilton et al., 2008) and in primary cultured hepatocytes of freshwater tilapia (Liu et al., 2007) exposed to PFOA, as well as in salmon hepatocytes exposed to perfluorooctane sulfonamide (PFOSA) (Olufsen and Arukwe, 2015). Similarly, a non-activation of GPx activity was known in Atlantic salmon hepatocytes exposed to PFOSA (Wågbø et al., 2012) and in hepatocytes of freshwater tilapia exposed to PFOA (Liu et al., 2007). This result can be explained by the fact that other components of the antioxidant system may have been activated. It is known, for example, that in the cell, in addition to CAT and GPx, the Prdxs, as mentioned above, also operate as H_2_O_2_ scavengers (Al-Asadi et al., 2019).

In contrast, the caudal kidney showed induction of both CAT and Se-GPx. This finding is expected, given that the kidney is the second most important organ for PFAS accumulation (Savoca and Pace, 2021), as also confirmed by our chemical analyses, which demonstrated increasing levels of PFOS and PFDA in fish moving from the control site to the highly-polluted one. Specifically, PFOS levels reached 70.54 μg/L and PFDA 32.90 μg/L in the caudal kidney of fish from the highly-polluted site. Similar trends of PFAS bioaccumulation, with PFOS predominance in various organs, have been reported in freshwater fish (Pivonkova et al., 2020; Smith et al., 2021). PFDA has been shown to induce oxidative stress and mitochondrial dysfunction in cellular and zebrafish embryo models, impairing fatty acid β-oxidation and increasing reactive oxygen species production (Widhalm et al., 2024; Piva et al., 2022). These effects support the mechanistic plausibility of the observed activation of antioxidant enzymes such as CAT and Se-GPx in the caudal kidney, as a defensive response to PFAS-induced oxidative stress. The complementary induction of these enzymes, varying across pollution levels, suggests a dynamic adjustment of the antioxidant system to the intensity of oxidative challenge.

In specimens sampled from the low-polluted site, there is a renal increase in Se-GPx activity but not in CAT. The opposite occurs in the medium-polluted site. Notably, antioxidant defences work in a complementary manner: Godin and Garnett (1992) previously correlated a low GPx activity with a high CAT activity; the opposite condition was pointed out in the liver of two Antarctic fish species, where high Se-GPx activity corresponded to lower CAT activity (Santovito et al., 2012). CAT typically plays a minor role at low H_2_O_2_ concentrations but becomes useful when this reactive oxygen species (ROS) increases (Regoli et al., 2005). Therefore, we can assume that Se-GPx has a first-line defence role against PFAS-induced oxidative stress, while CAT activity is enhanced when the risk of oxidative stress increases. In the highly-polluted site, both enzymes’ high activities were expected as ROS production could be so high that all antioxidant defences are required to counteract oxidative stress. The cooperation of different antioxidant system components is crucial, especially if the studied fish come from an environment subjected to viability in PFAS concentrations over time, so they must be ready to counteract future PFAS damages effectively.

The expressions of *cat*, *gpx-1*, and *gpx-4* mRNAs were measured in the liver and caudal kidney and compared among the four sampling sites, whether polluted or not by PFAS, and with the amount of the relative active proteins for each site.

Another interesting feature of the activation of the antioxidant system in *S. cephalus* exposed to PFAS is the mismatch between the expression profiles at the gene (mRNA) and protein (enzyme activities) levels, which is not directly attributable to the different half-lives of the respective molecules. The absence of correlation between gene transcription and messenger translation referring to anti-stress proteins, such as those of the antioxidant system, was documented in our previous works in aquatic organisms (Ferro et al., 2013; Drago et al., 2022), including fish (Sattin et al., 2015; Tolomeo et al., 2019; Bakiu et al., 2022b; Pacchini et al., 2023). Many authors attribute this phenomenon to a post-transcriptional control on protein synthesis operated by stress granules (SGs) (Takahashi et al., 2013; Harvey et al., 2017; Curdy et al., 2021; Drago et al., 2021; Bakiu et al., 2024). These non-membranous cytoplasmic foci operate in the recruitment of silenced mRNAs due to the presence of specific mRNA-binding proteins (Drago et al., 2021; 2023; Piva et al., 2024). The mechanism by which these proteins operate in mRNA selection within SGs remains a subject of study (Khong et al., 2017; Glauninger et al., 2024). However, SG-based regulation could explain why increased CAT and Se-GPx activity in the caudal kidney did not match increased mRNA expression for the *cat* and *gpx-1* genes. Indeed, the presence of PFAS-induced oxidative stress could induce the disassembly of SGs, leading to the release of specific mRNAs contained therein, which unlocks their translation and facilitates the biosynthesis of antioxidant enzymes.

The potential interaction between SGs and the antioxidant system requires future confirmation, and investigations into this effect are already underway. What is already evident is that not all components of the antioxidant system are subject to such regulation. Indeed, in the caudal kidney, our data indicate that the *gpx-4* expression profile perfectly correlates with Se-GPx activity, thus suggesting that this isoform makes the main contribution to enzyme formation. Like other GPx isoforms, GPx-1 is predominantly expressed inside the cytoplasm, whereas GPx-4 is the only one present inside mitochondria (Pei et al., 2023), which are considered one of the PFAS intracellular toxicity targets (Hagenaars et al., 2013). Therefore, GPx-1 is probably not the most essential isoform in response to the stress caused by these pollutants.

## 5 Conclusions

The results obtained in this study provided valuable insights into the physiological strategies adopted by *S. cephalus* to survive in PFAS-contaminated rivers in the Veneto region, highlighting its potential as a model organism for assessing the impact of PFAS on European freshwater fauna. These strategies involve activating the antioxidant system in the caudal kidney, particularly Se-GPx and CAT, and the accumulation of lipids in the liver and caudal kidney to reduce the reactivity of PFAS to their target molecules, i.e., proteins. This study highlights the importance of monitoring the riverine fauna in its ability to acclimatise to an environment where human impact is becoming increasingly important.

There is certainly still much to evaluate and improve, such as the analysis of PFAS accumulation in different organs and tissues, which remains hindered by numerous methodological limitations.

Future research will expand the investigation of PFAS accumulation and anti-stress responses to a broader range of organs, including the brain, in addition to the kidney and liver analysed in the present study, despite the persistent methodological challenges in accurately quantifying tissue PFAS concentrations. The effects of PFASs on the brain are little known in fish. Still, it appears that they may alter brain function by interfering with amino acid neurotransmitter metabolism and disrupting blood-brain barriers (Wang R. et al., 2023), thus influencing the behaviour and inducing stress in these animals, similarly to other environmental perturbations (Schumann et al., 2023; 2025). Particular focus will be placed on histological confirmation of the inflammatory condition in the spleen, aiming to shed light on the development of specific pathologies resulting from chronic stress induced by PFAS, which may be a consequence of constant ROS production (Lushchak, 2016). In this respect, it will also be necessary to evaluate the role played by other components of the antioxidant system that are often overlooked, such as the aforementioned Prdxs or methionine sulphoxide reductase, which plays a key role in repairing oxidative damage (Ricci et al., 2017). And of course, the oxidation levels of lipids and proteins to better understand whether this new steady state of ROS is actually kept under control by the antioxidant system of *S. cephalus* (Piva et al., 2022) and, therefore, whether these fish will be able to survive a possible future increase in the environmental concentration of PFAS.

## Data Availability

The datasets presented in this study can be found in online repositories. The names of the repository/repositories and accession number(s) can be found below: https://researchdata.cab.unipd.it/id/eprint/1627, Research Data Unipd repository.

## Abbreviations

ANOVA, one-way variance analysis; ARPAV, regional agency for environmental prevention and protection of Veneto; CAT, catalase; CDS, coding DNA sequence; CE, collision energy; CV, cone voltage; FCF, Fulton’s condition factor; GPx, glutathione peroxidase; GSH, reduced glutathione; HSI, hepatosomatic index; LOD, limit of detection; LOQ, limit of quantification; PFAS, per- and polyfluoroalkyl substances; PFDA, perfluorodecanoic acid; PFOA, perfluorooctanoic acid; PFOS, perfluorooctane sulfonate; PFOSA, perfluorooctane sulfonamide; Prdx, peroxiredoxin; QC, quality control; qRT-PCR, quantitative real-time PCR; ROS, reactive oxygen species; Se, selenium; SGs, stress granules; SSI, spleen somatic index; UHPLC-MS/MS, ultra-high-performance liquid chromatography coupled with tandem mass spectrometry.
