# Supplementary material for: From genes to organs: physiological responses of European chub (Squalius cephalus) to chronic PFAS pollution
Source: Front Toxicol. 2025 Sep 26;7:1654272. doi: 10.3389/ftox.2025.1654272 (PMC12511053; doi:10.3389/ftox.2025.1654272)
Supplement: Supplementary file 1 [file Supplementaryfile1.docx]

**Supplementary materials (Figure S1 and Tables)**


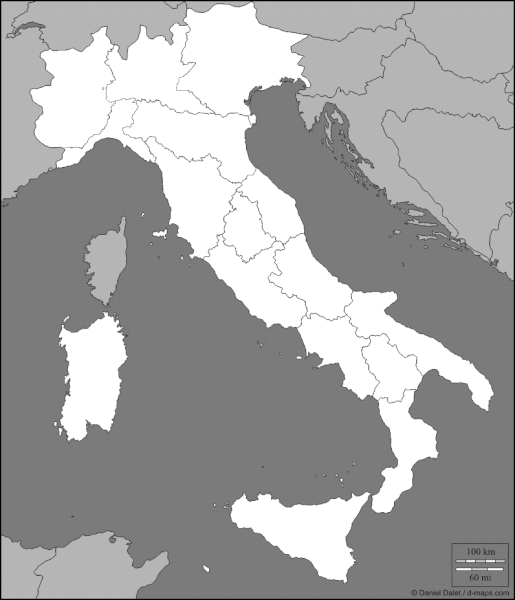


**Figure S1.** Map of the study area in the Veneto region (Vicenza province), showing Roggia Moneghina as control site, Fosso Brenta as low-polluted site, Scolo Togna as medium-polluted site, and Torrente Poscola as highly-polluted site, indicated by green, yellow, orange, and red arrows, respectively. Gauss-Boaga coordinates of the sites are reported in Table S1.

**Table S1.** Total PFAS average concentration in freshwater from the four sampling sites, measured by ARPAV in 2022 and 2023. The Gauss-Boaga coordinates, obtained with qGIS (version 3.22) software, are reported for the geo-localisation of the sites.

| **Sampling sites** | **Gauss-Boaga coordinates** | **Total PFAS average concentration (ng/l)** |
| --- | --- | --- |
| Control site  (Roggia Moneghina) | X: 1707494.771; Y: 5043242.570 | < 10 |
| Low-polluted site  (Fosso Brenta) | X: 1688840.326; Y: 5047241.734 | 185.263 |
| Medium-polluted site  (Scolo Togna) | X: 1685003,474; Y: 5029108,091 | 855.704 |
| Highly-polluted site  (Torrente Poscola) | X: 1686344.594; Y: 5046652.211 | 1116.14 |

**Table S2.** Primer sequences used for qRT-PCR. For all the primers, 60°C was used as the annealing temperature.

| **Primers** | **Sequences 5’-3’** |
| --- | --- |
| CAT_RT_forward | GCGTCCTGAATCGTTGCACCA |
| CAT_RT_reverse | TGACCCTCAGCGTTGACCAGT |
| GPx1_RT_forward | TCCATTCCCATTCGATGACCCAAT |
| GPx1_RT_reverse | GGCACTCCATCAGAACCGATAAGA |
| 1_FW_GPx4_CAV_RT | AGCGGACATAAAGGAGTTTGCT |
| 1_RV_GPx4_CAV_RT | CCTCTTCACGACCCGACCTT |
| sq_GAPDH_b_fw | ATCACAGCCACACAGAAGAC |
| sq_GAPDH_b_rv | AGGAATGACTTTGCCCACAG |

**Table S3.** Optimized mass transitions for PFAS and their internal standards, with corresponding cone voltage and collision energy. The first transition was used for quantification and the second for confirmation.

| **Analyte** | **MRM transitions (*m/z*)** | **Cone voltage (V)** | **Collision energy (eV)** |
| --- | --- | --- | --- |
| PFBA | 212.0 > 169.0 | 8 | 7 |
|  | 212.0 > 64.7 | 8 | 22 |
| PFBS | 299.0 > 79.9 | 22 | 28 |
|  | 299.0 > 98.9 | 22 | 28 |
| PFPeA | 263.0 > 219.0 | 6 | 7 |
|  | 263.0 > 69.0 | 6 | 32 |
| PFHxA | 313.0 > 269.0 | 10 | 7 |
|  | 313.0 > 118.9 | 10 | 18 |
| PFHxS | 399.0 > 79.9 | 20 | 36 |
|  | 399.0 > 98.9 | 20 | 34 |
| PFHpA | 363.0 > 319.0 | 15 | 8 |
|  | 363.0 > 169.0 | 15 | 16 |
| PFOA | 412.9 > 369.0 | 15 | 9 |
|  | 412.9 > 169.0 | 15 | 18 |
| PFOS | 498.0 > 98.9 | 30 | 40 |
|  | 498.0 > 79.9 | 30 | 45 |
| PFNA | 463.0 > 418.9 | 20 | 9 |
|  | 463.0 > 218.9 | 20 | 15 |
| PFDA | 512.8 > 469.0 | 20 | 10 |
|  | 512.8 > 218.9 | 20 | 17 |
| GenX | 285.0 > 169.0 | 12 | 7 |
|  | 285.0 > 118.9 | 12 | 28 |
| ^13^C_2_-PFOA | 415.0 > 370.0 | 20 | 9 |
| ^13^C_4_-PFOS | 503.0 > 80.0 | 20 | 9 |
| ^13^C_3_-GenX | 286.0 > 169.0 | 12 | 9 |

**Table S4.** Concentrations of different PFAS compounds were detected in the organs of *S. cephalus* from the four sampling sites (the total environmental PFAS average concentration, as referenced in Table S1, has been reported). Due to insufficient tissue availability, samples from 8 specimens were pooled. Dashes (-) indicate PFAS not detected.

| SITES AND TOTAL PFAS | ORGANS | PFOS  (μg/l) | PFOA  (μg/l) | PFDA  (μg/l) | PFNA  (μg/l) | PFHxS  (μg/l) | PFBS  (μg/l) |
| --- | --- | --- | --- | --- | --- | --- | --- |
| CONTROL  (< 10 ng/l) | Liver | 7.58 | - | 4.00 | - | - | - |
|  | Caudal kidney | 14.29 | - | 5.38 | - | - | - |
| LOW-POLLUTED  (185 ng/l) | Liver | - | 0.43 | 2.24 | - | - | - |
|  | Caudal kidney | - | 1.33 | 4.11 | - | - | - |
| MEDIUM-POLLUTED  (856 ng/l) | Liver | 55.40 | 0.36 | 3.31 | 0.062 | 0.95 | 0.067 |
|  | Caudal kidney | 41.87 | 1.84 | 10.12 | 0.25 | 0.52 | 0.030 |
| HIGHLY-POLLUTED  (1116 ng/l) | Liver | 72.78 | 0.64 | 21.67 | - | 0.55 | 0.15 |
|  | Caudal kidney | 70.54 | 0.96 | 32.90 | - | - | - |
